# COVID-19: Pulmonary and Extra Pulmonary Manifestations

**DOI:** 10.3389/fpubh.2021.711616

**Published:** 2021-09-28

**Authors:** Islam H. Elrobaa, Karl J. New

**Affiliations:** ^1^Emergency Medicine Specialist in Hamad Medical Corporation, Qatar and Lecturer in Clinical Education Department, College of Medicine, Qatar University, Doha, Qatar; ^2^Clinical Physiology, School of Health, Sport, and Professional Practice, Faculty of Life Science and Education, University of South Wales, Treforest, United Kingdom

**Keywords:** COVID-19, manifestations, pulmonary, extra pulmonary, infection control

## Abstract

**Introduction:** The coronavirus disease-2019 (COVID-19) pandemic has been the most significant event in 2020, with ~86.8 million cases and 1.88 million deaths worldwide. It is a highly infectious disease, wherein the virus (severe acute respiratory syndrome coronavirus 2) rapidly multiplies and spreads to all parts of the body. Therefore, COVID-19 is not only respiratory disease but also a multisystem disease. Many people, including physicians, incorrectly believe that the disease affects only the respiratory tract. In this study, we aimed to describe COVID-19 manifestations and the underlying pathophysiology to provide the readers with a better understanding of this disease to achieve good management and to control the spread of this disease.

**Methods:** Secondary data were obtained from PubMed, Google Scholar, and Scopus databases. The keywords used for the search were as follows: COVID-19, COVID-19 pulmonary manifestations, COVID-19 extra pulmonary manifestations, and pathophysiology of COVID-19. We collected secondary data from systemic reviews, metaanalyses, case series, and case reports in the form of public data that was published on websites of the government, medical corporations, medical peer-reviewed journals, and medical academies, all of which were indexed in PubMed, Google Scholar, or Scopus. Our questions were as follows: Is COVID-19 a respiratory disease only? and What are the extrapulmonary manifestations of COVID-19?

**Results:** From our data, we found that a patient with COVID-19 may be either asymptomatic or symptomatic. Symptomatic cases may have either pulmonary or extrapulmonary manifestations. Pulmonary manifestations occur as mild, moderate, or severe cases. In mild and moderate cases, extrapulmonary manifestations such as gastroenteritis, fever, or vomiting may present alone. Some of these cases may be missed for diagnosis, and the patient may receive symptomatic treatment without a COVID-19 diagnosis, leading to increased spread of the infection. Extrapulmonary manifestations may occur in severe and critical cases as complications of severe infections (high viral overload) or the cytokine storm, such as in acute kidney injury (AKI), heart failure (HF), and venous thromboembolic (VTE) manifestation.

**Conclusion:** COVID-19 is not a respiratory disease alone; rather, it is a multisystem disease. Pulmonary and extrapulmonary manifestations should be considered for early diagnosis and to control the spread of the infection.

## 1. Introduction

The novel coronavirus disease-2019 (COVID-19) is currently one of the most rapidly spreading diseases worldwide. The causative pathogen, severe acute respiratory syndrome coronavirus 2 (SARS-CoV-2), is an animal and a human pathogen. The disease originated in Wuhan City, China, and then spread to the rest of the world (1). The chief features of the disease are as follows: (1) a high viral-multiplying capacity, (2) an extensive spread leading to a high prevalence, (3) being a newly discovered novel disease, there are no standardized treatment regimens until recently, and (4) a high mortality rate in some communities (2).

The disease may present as mild, moderate, or severe in terms of the severity of presentation. The mild disease may be characterized by symptoms such as body aches, coughs, or mild fever, while in its moderate form the disease may present with mild pneumonia along with other symptoms. The severe form of the disease presence may be characterized by severe pneumonia and hypoxia. Critical cases with significant hypoxia and organ failure may need admission to the intensive care unit (ICU) and mechanical ventilation support (3–5). Besides the symptomatic cases, asymptomatic cases have also been reported (5–7).

Due to the rapid spread of the disease worldwide, many countries were forced to close their borders and impose internal lockdowns to curb the spread. With an incubation period of 14 days (2), the symptoms can be detected ~4–5 days after exposure (4). Currently, the mortality rate due to the disease is ~2–5% according to the community, but it may reach as high as 7% as observed in Italy (8–10).

Severe and critical forms can be easily identified based on the presence of SARS symptoms (2) and confirmed with chest radiography (5). The mild and moderate forms may have non-specific symptoms such as fever, gastroenteritis, vomiting, dysgeusia (loss of taste), and headache with no or mild respiratory symptoms (11). Knowledge of these extrapulmonary manifestations can help in detecting the mild and moderate forms, which can aid in early diagnosis, and rapid quarantining can prevent community spread.

In this review, we have discussed COVID-19 as both a respiratory tract infection and a multisystem disease. We also discuss the underlying pathophysiology of the disease and its manifestations, which can serve as a basis for good management, treatment, and infection control. In addition, the results of this study will provide an understanding of the COVID-19, enabling our medical colleagues to improve the quality of health care management.

## 2. Methods

We collected data on COVID-19 from the databases PubMed, Google Scholar, and Scopus. We used the following keywords in our search: COVID-19 pulmonary manifestations, extrapulmonary manifestations of COVID-19, COVID-19 clinical presentations, the pathophysiology of COVID-19, and management of COVID-19. We collected secondary data from systemic reviews, meta-analyses, case series, and case reports from public data published on websites of the government, medical corporations, medical peer-reviewed journals, and medical academies, which were all indexed on PubMed, Google Scholar, or Scopus. We collected data about the clinical manifestations, pathophysiological effects, and the management of the COVID-19 disease. Specific questions were raised to understand the presentations and manifestations of COVID-19. Does COVID-19 present exclusively as pulmonary manifestations only? What are the extrapulmonary presentations of COVID-19? Complete understanding of COVID-19 in terms of its pathophysiology and manifestations leads to good planning regarding the control and prevention of the spread of the disease.

## 3. Results

**Figure 1:**
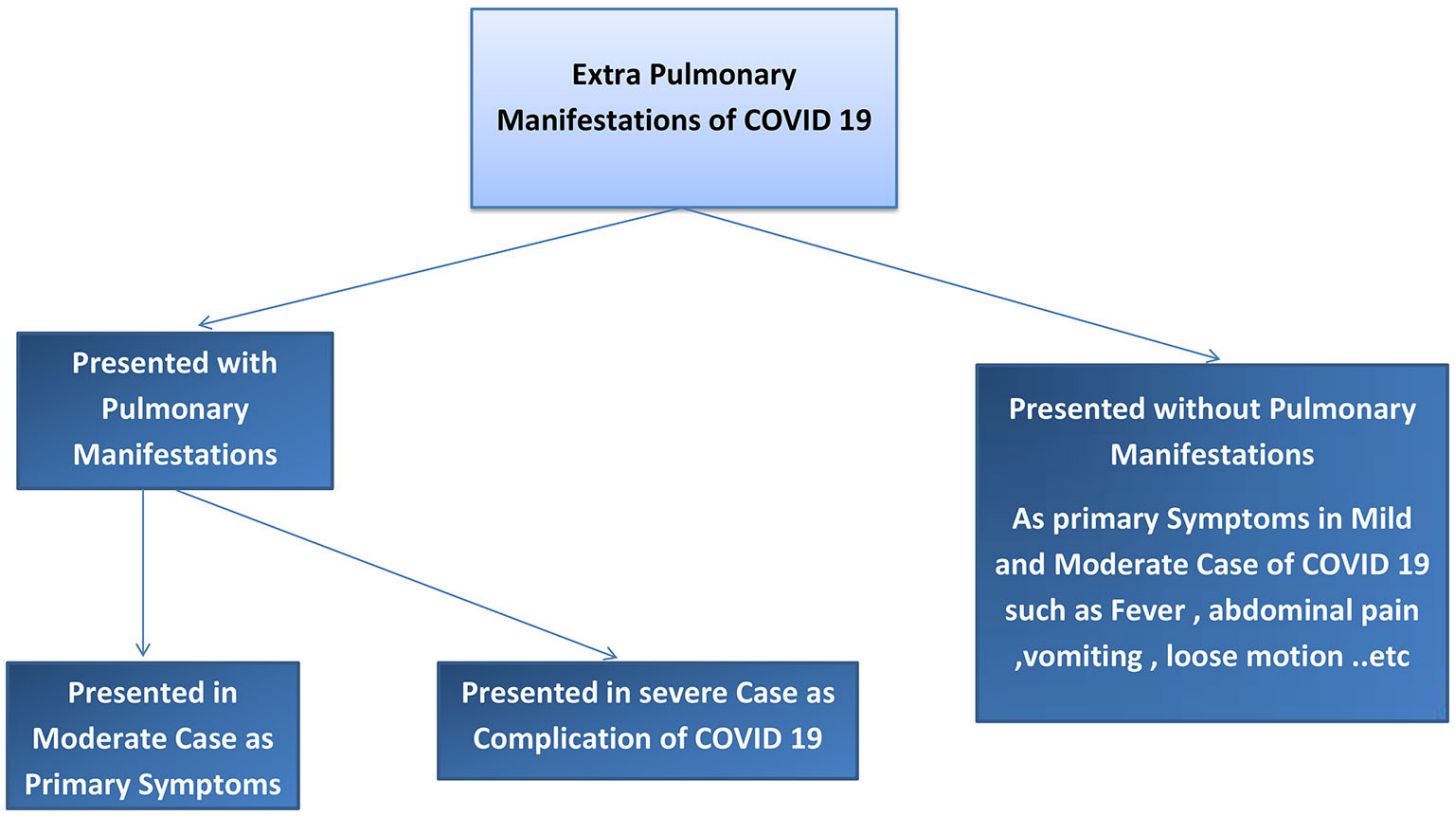
Diagram to explain the presentation of extrapulmonary manifestations of COVID-19.

**Figure 2:**
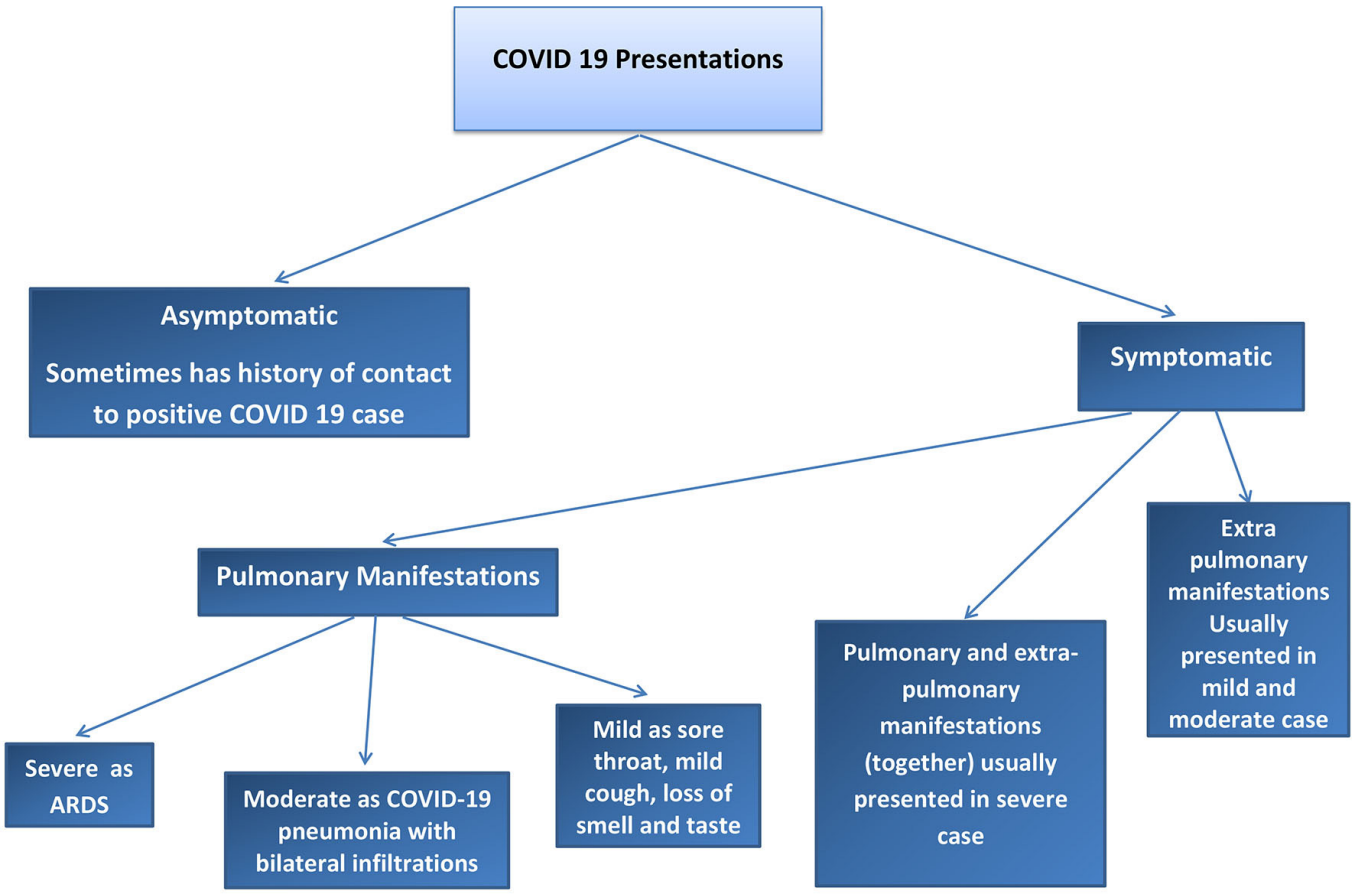
Diagram to explain the COVID-19 presentations in Health care provider centres.

We found two main types of COVID-19 manifestations: pulmonary and extrapulmonary manifestations. Pulmonary manifestations are most well-known because these cases are critical, difficult to manage, and have poor outcomes. Extrapulmonary manifestations of COVID-19 are common in moderate and mild cases and may also occur together with pulmonary manifestations or in severe infection cases, with multiple complications. Many physicians may not be sufficiently aware of these extrapulmonary manifestations; therefore, such cases may be missed to be identified and can cause the spread of COVID-19 to a greater degree in the community. Asymptomatic cases of COVID-19 have also been reported. To understand COVID-19 manifestations, we should understand the underlying mechanisms and pathophysiology of the disease. Risk factors that can determine COVID-19 progression in the patient should be considered. The pathophysiology of COVID-19 may help in understanding how to treat and manage this disease. Our results explain the pathophysiology of COVID-19, risk factors that determine the COVID-19 effect and spread, pulmonary and extrapulmonary manifestations of COVID-19, and the management of COVID-19. Refer to Figures 1, 2.

### 3.1. Pathophysiology of COVID-19

Understanding disease pathophysiology is crucial to understand the clinical presentation. The coronavirus (CoV) is classified according to its genotypic form into alpha α, beta β, gamma γ, and omega o (12). α and β CoVs are the human infecting forms. SARS-CoV1 and SARS-CoV2 are members of β CoVs, but SARS-CoV2 has a spike of glycoprotein with a high affinity to the angiotensin-converting enzyme-2 (ACE2) receptors (13, 14). ACE2 receptors are located in the pulmonary alveolar cell type II, small intestine, colon, gallbladder, testes, brain stem, esophagus, heart, and blood vessels in the basal layer of the epidermis (15–17). This highlights the relation between the disease symptoms and ACE2 receptor distributions in the human body (7, 18, 19).

When blood pressure decreases in the renal juxta-glomerular apparatus, it releases renin into the bloodstream. Renin acts on angiotensin (from the liver) and converts it to angiotensin-I. Angiotensin-1, in turn, is converted into angiotensin-2 by the action of the angiotensin-converting enzyme (ACE1). Angiotensin-2 has a vasoconstrictor effect that increases blood pressure by increasing renal perfusion and stimulating aldosterone release (20).

Angiotensin-converting enzyme-2 has a contrary effect of ACE1, thereby reducing serum levels of angiotensin-2 (21). ACE2 binds to its receptor and catalyzes the hydrolysis of angiotensin II to angiotensin. This reduces blood pressure, heart rate, and alveolar surface tension, which is important for treating acute respiratory distress syndrome (ARDS) (22). ACE2 is the entry point for SARS CoV-2 into cells through endocytosis. The virus, along with the enzyme, is then translocated into the cell endosome (23).

Coronavirus disease-2019 spreads through physical contact and inhalation of infected droplets or air. It then invades the airway epithelium, where the viral load is increased by replication. Pyroptosis occurs by the leaking of the virus from the upper respiratory tract vasculature into the bloodstream, which then travels to other target organs that have ACE2 receptors. This process induces a T-cell-mediated inflammatory response, which releases interleukins and cytokines (24). This cytokine storm may lead to the development of ARDS (25).

The leaked SARS-CoV-2 then travels to other organs with ACE2 receptors, leading to extrapulmonary features, such as gastroenteritis (small intestine); insomnia, dysgeusia, and headache (brainstem, cerebral cortex, and hypothalamus, respectively), high blood pressure, and tachycardia (heart and blood vessels, respectively), and some skin infections (basal epidermis). ACE2 receptors may be present in the retina and other eye tissues, leading to conjunctivitis when infected (26, 27). A host of other immune and inflammatory responses are also triggered by the infection (15, 28–33).

Many factors can determine the severity of COVID-19, including viral load, genetic factors, presence of comorbidities, age, sex, use of immune-suppressive agents, and immunity (34). Some authors believe that genetic factors also play a role in the severity of COVID-19 because the ACE2 receptor gene has multiple polymorphisms, which means that there are multiple variations in the relationship between SARS-CoV-2 and ACE2 receptor according to ethnicity and gene form. This difference in a relationship can explain the severity of the disease in a specific race than others (35).

People with comorbidities such as diabetes mellitus, hypertension, chronic obstructive pulmonary disease (COPD), chronic lung disease, chronic kidney disease, cancer, and low immunity may have a greater affinity for severe infections and serious complications of COVID-19 (7). Many comorbidities lead to defects in ACE2 expression (36). For example, patients with essential hypertension may have defects in the ACE1/ACE2 balance, which leads to severe symptoms of COVID-19 in that patient. Some studies have suggested that patients with comorbidities have a more severe form of COVID-19, and studies that included a high proportion of patients with ARDS as a complication of COVID-19 showed that the patients had comorbidities before contracting the infection (37).

Some studies have shown that elderly people have a greater disease severity than young people (38). Most pulmonary complications, such as ARDS, are more frequent in the elderly with COVID-19, and multiple organ failures also occur with a higher frequency in elderly people with COVID-19 (39). Immunity and comorbidity are predisposing factors that affect the complications of COVID-19 in the elderly (40). Some studies have suggested that ACE2 expression is higher in the lungs of old people than in the lungs of people of other ages (41). In addition, the mortality rate is higher in the elderly group (42).

Many studies have suggested that men are affected more by COVID-19 than women because the ACE2 expression is higher in men. Furthermore, the testes may also express ACE2, and ACE2 levels in the plasma are higher in men than in women. Some lifestyle factors associated with COVID-19 were smoking, alcohol consumption, and other factors that are more common in men than in women. Other studies have shown that critical care admission was higher in men with COVID-19 than in women with COVID-19. The mortality rate in men with COVID-19 was also higher than that in women with COVID-19 (43, 44).

Obesity plays a role in COVID-19 severity because ACE2 expression is higher in adipose tissue (45). Obese people have a higher risk of respiratory failure and are more frequent candidates for mechanical ventilation (46). Many COVID-19 critical cases have a higher body mass index (47). The mortality rate with COVID-19 is higher in obese patients because of respiratory failure and heart failure [HF; (48)]. Obese people may also have more comorbidities such as hypertension and diabetes mellitus, factors that affect COVID-19 severity (49). In essential hypertension, there is a defect in the ACE/ACE2 balance, and this is more common in obese patients (50).

#### 3.1.1. Cytokine Storm

The physiological immunological reaction by the innate immune system can cause an excessive release of proinflammatory cytokines such as interleukins and cytokines (51). The cause may be cytomegalovirus and streptococcal A infection or a skin graft. The excessive release of cytokines and interleukins may lead to a huge inflammatory process in the body that induces multiorgan failure and ARDS (52).

**Figure 3:**
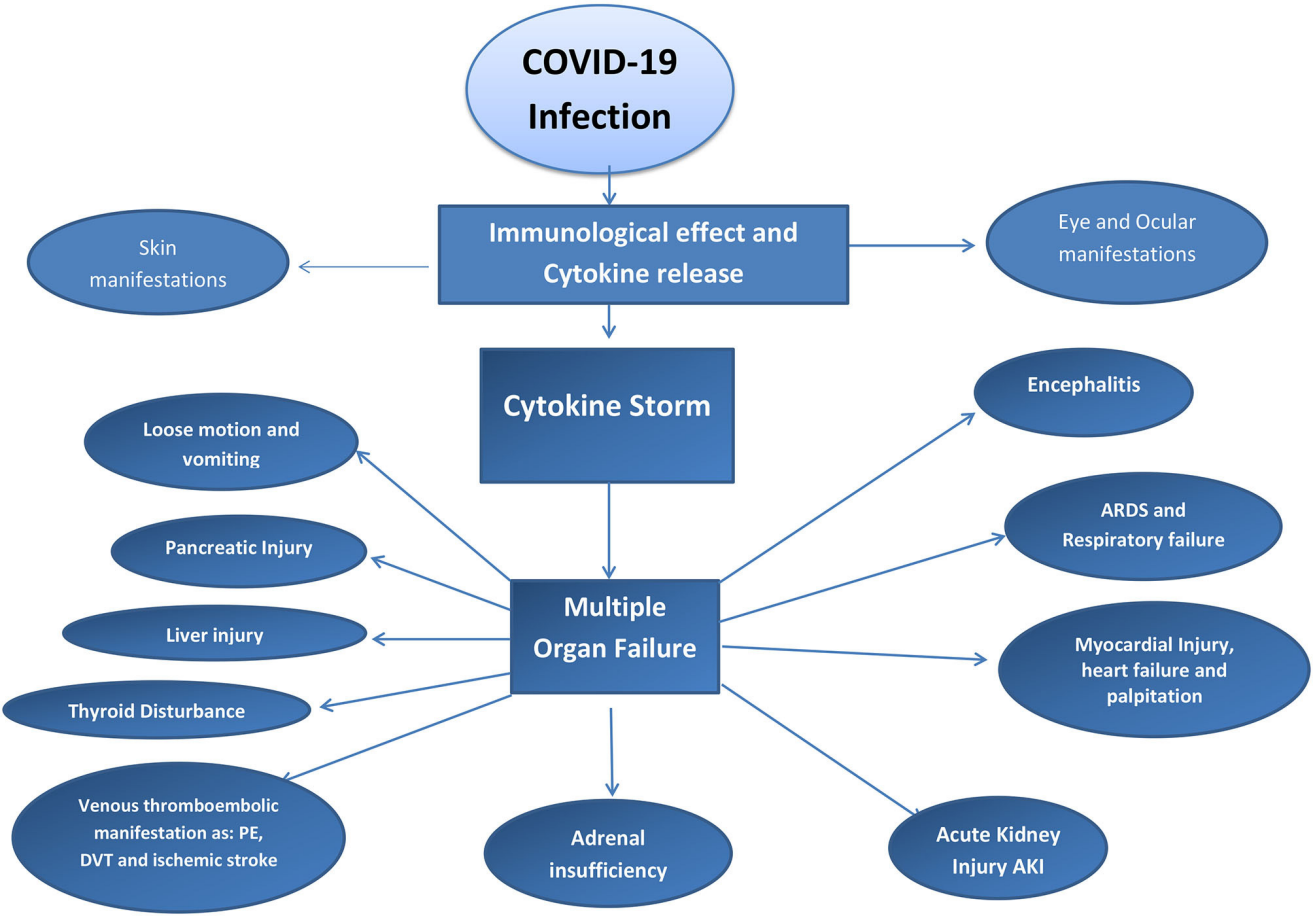
Diagram to explain Cytokine storm effect in COVID-19 patient.

Immunity plays a major role in determining the severity and reactions of COVID-19 (53). Some authors have mentioned that people with low immunity are at a greater risk for severe respiratory distress and multiple organ failure due to COVID-19 (54). In contrast, many studies reported that a high immune reaction can predispose a cytokine storm that leads to acute respiratory distress and multiple organ failure by immune-mediated reactions against the body tissue and organs. Acute kidney injury (AKI), ARDS, myocarditis, skin manifestations, ocular manifestations, and neurological manifestations of COVID-19 may occur due to the cytokine storm (55). Immunosuppressive agents should not be stopped in patients who are being treated with these agents. Many studies have suggested that patients who use cytokine inhibitors do not exhibit the severe features of COVID-19 because of the inhibitory action on the hyperinflammatory condition of COVID-19 (56). Refer to Figure 3.

### 3.2. Pulmonary Manifestation of COVID-19

Pulmonary manifestations in COVID-19 may be mild, moderate, and severe (57). Mild cases such as upper respiratory tract infection (URTI), cough, or sore throat can progress to moderate and severe degrees (58). The moderate type of pulmonary presentation of COVID-19 could be pneumonia and fever (59). The COVID-19 pneumonia type has been reported in some cases as silent pneumonia with fever or silent pneumonia in a sick patient (60). We found cases of silent hypoxia as a result of silent COVID-19 pneumonia (60). The severe manifestation of COVID-19 is ARDS (61). There are two pathological mechanisms to explain ARDS in COVID-19 patients. The first mechanism discusses that the ACE2 receptor is located in pneumocyte type II (62). ACE2 and pneumocyte type 2 play a role in producing the pulmonary surfactant (62, 63). ACE2 can improve the blood flow in the alveolar cell, which improves the alveolar cell function as a pneumocyte type 2, which produces the pulmonary surfactant (62, 63). The ACE2 receptor is the host of SARS-COV-2 to the human cell. So when SARS-CoV-2 binds to ACE2 receptors, it leads to destruction and damage in the alveolar cells and decreases the pulmonary surfactant that leads to an increase in the surface tension of the lung and predisposition to ARDS (62, 63). The second suggested mechanism for ARDS in COVID-19 patients is the cytokine storm. Cytokine storm is a hyperimmune response to specific triggers such as viral infections that lead to a large release of proinflammatory cytokines, such as cytokines and interleukins, leading to an excessive inflammatory response that leads to multiple organ failure and destruction in alveolar cells with ARDS. Multiple factors can determine the severity of the pulmonary manifestations such as viral overload, genetic roles and ethnicity, presence of comorbidities, age, and sex (64). The most common causes of mortality in COVID 19 are ARDS, severe COVID-19 pneumonia, and multiple organ failure (65). Refer to Table 1.

**Table 1:** Pulmonary manifestations of COVID 19.

**Manifestations**	**References**	**Patients**	**The affected patients**	**Incidence**
**URTI** **Pneumonia and Fever**	**Liu, Ping et al. “Epidemiological and clinical features in patients with coronavirus disease 2019 outside of Wuhan, China: Special focus in asymptomatic patients.” PLoS neglected tropical diseases vol. 15,3 e0009248. 10 Mar. 2021, doi:** 10.1371/journal.pntd.0009248	**209**	**21** **180**	**10 %** **86.1%**
**Silent Hypoxia**	**Teo, Jason. “Early Detection of Silent Hypoxia in Covid-19 Pneumonia Using Smartphone Pulse Oximetry.” Journal of medical systems vol. 44,8 134. 19 Jun. 2020, doi:** 10.1007/s10916-020-01587-6	**—–**	**——-**	*Seen frequently in our Practicein HMC, Qatar
	**Wilkerson, R Gentry et al. “Silent hypoxia: A harbinger of clinical deterioration in patients with COVID-19.” The American journal of emergency medicine vol. 38,10 (2020): 2243.e5-2243.e6. doi: ** 10.1016/j.ajem.2020.05.044	**Case report**	**Case report**	
**ARDS**	**Tzotzos, Susan J et al. “Incidence of ARDS and outcomes in hospitalized patients with COVID-19: a global literature survey.” Critical care (London, England) vol. 24,1 516. 21 Aug. 2020, doi:** 10.1186/s13054-020-03240-7	**2212**	**738**	**33%**

#### 3.2.1. Discussion Point

Pulmonary manifestations in COVID-19 may present as URTI and cough progressing to ARDS or as a sudden stack of ARD due to silent pneumonia and hypoxia. Sometimes, a bilateral ground-glass appearance can be seen on a CT scan for asymptomatic cases of COVID-19. The most common features of the chest radiographic manifestations of COVID-19 are bilateral infiltration, bilateral ground-glass appearance, bilateral pneumonia (patches), and pleural effusion. However, the American College of Radiology recommends not to perform a CT scan to diagnose COVID-19 in asymptomatic patients (Refer to below section COVID-19 Manifestations and Diagnosis).

### 3.3. Cardiovascular Manifestations of COVID-19

Angiotensin-converting enzyme receptors are located in the cardiac tissue, and during a SARS-CoV-2 attack, they may cause cardiac tissue degeneration because the ACE receptors are affected by SARS-CoV-2. Many cardiac manifestations have been reported in COVID-19 cases such as arrhythmias, hypertension, palpitations, myocarditis, myocardial injuries, cardiomyopathies, and HF (66). Two mechanisms are leading to the response of cardiac manifestations of COVID-19. The first is the effect of SARS–CoV-2 on the ACE2 receptors in the cardiac tissue. The second mechanism is the cytokine storm that can cause HF and other cardiac manifestations (67). Viral overload sometimes determines the severity of the extrapulmonary manifestations, such as cardiovascular symptoms of COVID-19 (68). Some of the medications that were used to treat COVID-19-induced arrhythmias included hydroxychloroquine and chloroquine (69). Some clinical studies have shown elevated levels of cardiac enzymes such as troponin T in ICU COVID-19 patients (70). Elevation in pro BNP levels was also reported in ICU patients. Some retrospective studies showed diffuse ST elevations in COVID-19 patients, and urgent coronary angioplasty showed no coronary obstruction (71). However, myocardial infarction (MI) was reported in the ICU and hospitalized COVID-19 patients (72). Some authors mentioned arrhythmias in COVID-19 patients admitted to the ICU; most of these arrhythmias were paroxysmal atrial fibrillation (73). One of the studies recorded sudden cardiac death in 19 hospitalized patients; the patients had an HF with a low ejection fraction. Another study mentioned Takotsubo cardiomyopathy as one of the COVID-19 cardiovascular complications (74). Hypertension and palpitations were reported in many COVID-19 patients in their first visit to the health care provider. Hypoxia was counted as the cause of the cardiac arrhythmias and type 2 MIs in COVID-19 patients. MI in COVID-19 patients may be presented as type I, which results in the rupture of plaque thrombus, leading to occlusion of the coronary artery, or may be a result of severe hypoxia that leads to an ischaemic type II MI (75). Refer to Table 2.

**Table 2:** Cardiovascular manifestations of COVID 19.

**Cardiovascular** **manifestations**	**References**	**Patients**	**Affected patients**	**Incidence**
**Arrhythmia**	**Wang, Dawei et al. “Clinical Characteristics of 138 Hospitalized Patients With 2019 Novel Coronavirus-Infected Pneumonia in Wuhan, China.” JAMA vol. 323,11 (2020): 1061-1069. doi: ** 10.1001/jama.2020.1585	**138**	**23**	**16%**
**Cardiac injury and** **high troponin T**	**Huang, Chaolin et al. “Clinical features of patients infected with 2019 novel coronavirus in Wuhan, China.” Lancet (London, England) vol. 395,10223 (2020): 497-506. doi: ** 10.1016/S0140-6736(20)30183-5	**41**	**5**	**12%**
**AF**	**Inciardi, Riccardo M et al. “Characteristics and outcomes of patients hospitalized for COVID-19 and cardiac disease in Northern Italy.” European heart journal vol. 41,19 (2020): 1821-1829. doi: ** 10.1093/eurheartj/ehaa388	**99**	**19 [ known cardiac disease ]**	**19 %**
**Sudden cardiac death**	**Baldi, Enrico et al. “COVID-19 kills at home: the close relationship between the epidemic and the increase of out-of-hospital cardiac arrests.” European heart journal vol. 41,32 (2020): 3045-3054. doi: ** 10.1093/eurheartj/ehaa508	**321 in 2019 And 490 in 2020**	**169**	**34 %**
**Takotsubo cardiomyopathy**	**Bottiroli M, De Caria D, Belli O, et al. Takotsubo syndrome as a complication in a critically ill COVID-19 patient. ESC Heart Fail. 2020. doi: ** 10.1002/ehf2.12912	**Case report**		
**Hypertension**	**Wang, Dawei et al. “Clinical Characteristics of 138 Hospitalized Patients With 2019 Novel Coronavirus-Infected Pneumonia in Wuhan, China.” JAMA vol. 323,11 (2020): 1061-1069. doi: ** 10.1001/jama.2020.1585	**138**	**43**	**31.2%**
**Type II MI**	**Shi S, Qin M, Shen B, et al. Association of cardiac injury with mortality in hospitalized patients with COVID-19 in Wuhan, China. JAMA Cardiol 2020; doi: ** 10.1001/jamacardio.2020.0950	**416**	**82**	**19.7%**

#### 3.3.1. Discussion Point

The cardiovascular manifestation of COVID-19 may present initial symptoms, such as palpations, new diagnoses of hypertension, or chest pains, and may also present as late symptoms or complications in the ICU or hospitalized COVID-19 patient. The cardiovascular manifestations were reported in young, middle-aged, and old age patients with COVID-19.

COVID-19 may induce the following: (1) hypoxia by ARDS that leads to decreased oxygen supply to the heart muscle, leading to myocardial degradation and increased troponin T levels that lead to type II MI, (2) vascular injury due to the effect of SARS-CoV-2 on endothelial cells that lead to platelet aggregation and vascular plaques or thrombi that on detachment block the coronary blood vessels and can induce ischaemic MI. (3) SARS-CoV-2 has direct effect on ACE2 receptors on the myocardium that can cause myocarditis. (4) The cytokine storm may lead to cardiomyopathy and HF. Over-disease stress may lead to Takotsubo cardiomyopathy. Many viral overloads and genetic factors play a role in determining the cardiovascular complications and their severity in COVID-19 patients.

### 3.4. Gastroenterology Manifestations

The COVID-19 patient may present with loose motion, anorexia, nausea, vomiting, abdominal pain, epigastric pain, liver injury, and pancreatic injury. Elevated levels of liver enzymes including ALT, AST, and alkaline phosphatase have been reported (76). In addition, high levels of lipase and amylase were reported in some patients with COVID-19 (77). The mechanism of Gastrointestinal Tract (GIT) manifestations in COVID-19 depends on three factors: viral overload, and the direct effect of SARS-CoV-2 on the ACE2 receptors on the epithelium of the esophagus or mucus layer in the stomach and small intestine (17). ACE2 is also expressed in the liver tissue (78). The cytokine storm is considered the third factor that determines GIT symptoms, and it can induce gastroenteritis symptoms, liver injury, and pancreatic injury (79, 80). One retrospective study in China showed that 75% of COVID-19 patients had diarrhea (81). Also, same study reported abdominal pain in COVID-19 patient presentation (81). On other hand, liver injury with an elevation in liver enzyme reported in COVID 19 patients who admitted in the ICU (82). Some authors have mentioned acute pancreatitis as a cytokine storm complication (83). Some drugs, such as antiviral drugs, are considered hepatotoxic and induce liver injury in COVID-19 patients (84). The comorbidities, for example, diabetes mellitus and lifestyle factors including alcohol consumption, have a role in determining the symptoms and degree of COVID-19 cases (85). Some studies discovered the SARS-CoV-2 in the feces of COVID-19 patients (86). However, another study reported the presence of SARS–CoV-2 in the epithelium of the esophagus and mucus of the stomach in COVID-19 patients by endoscopy. However, endoscopy is safe in cases of GIT bleeding in COVID-19 cases (87). Refer to Table 3.

**Table 3:** GIT manifestations.

**GIT manifestations**	**References**	**Total patients**	**Affected patients**	**Incidence**
**Loose motion**	**Wan Y et al., 2020. Enteric involvement in hospitalised patients with COVID-19 outside Wuhan. Lancet Gastroenterol Hepatol 5: 534–535**	**230**	**49**	**23%**
**Vomiting, nausea and anorexia**	**Wu, Yi-Chi et al. “The outbreak of COVID-19: An overview.” Journal of the Chinese Medical Association : JCMA vol. 83,3 (2020): 217-220. doi: ** 10.1097/JCMA.0000000000000270	**80**	**1**	**1.25 %**
	**Wang, Dawei et al. “Clinical Characteristics of 138 Hospitalized Patients With 2019 Novel Coronavirus-Infected Pneumonia in Wuhan, China.” JAMA vol. 323,11 (2020): 1061-1069. doi: ** 10.1001/jama.2020.1585	**138**	**Vomiting in 5 patients** **Nausea in 14 patients**	**3.6%** **10.1%**
**Abdominal pain**	**Wang, Dawei et al. “Clinical Characteristics of 138 Hospitalized Patients With 2019 Novel Coronavirus-Infected Pneumonia in Wuhan, China.” JAMA vol. 323,11 (2020): 1061-1069. doi: ** 10.1001/jama.2020.1585	**138**	**3**	**2.2 %**
**Epigastric pain**	**Han, Chaoqun et al. “Digestive Symptoms in COVID-19 Patients With Mild Disease Severity: Clinical Presentation, Stool Viral RNA Testing, and Outcomes.” The American journal of gastroenterology vol. 115,6 (2020): 916-923. doi: ** 10.14309/ajg.0000000000000664	**206**	**9**	4.4%*seen frequently in our Practice in HMC, Qatar
**Pancreatic Injury**	**Liu F, Long X, Zhang B, Zhang W, Chen X, Zhang Z, 2020. ACE2 expression in pancreas may cause pancreatic damage after SARS-CoV-2 infection. Clin Gastroenterol Hepatol 18: 2128–2130.e2**.	**121**	**Amylase increased in 13 patients Lipase increased in 12 patients**	**10.74%** **9.92%**
**Liver injury**	**Fang D, Jingdong MA, Guan J, Wang M, Song J, Tian D, Peiyuan LI, 2020. Manifestations of digestive system in hospitalized patients with novel coronavirus pneumonia in Wuhan, China: a single-center, descriptive study. Chin J Dig 40: E005**	**99**	**43**	**43%**
**GIT bleeding**	**Cavaliere, Kimberly et al. “Management of upper GI bleeding in patients with COVID-19 pneumonia.” Gastrointestinal endoscopy vol. 92,2 (2020): 454-455. doi: ** 10.1016/j.gie.2020.04.028	**Case series of 6 patients with COVID-19 pneumonia and upper GI bleeding**		

#### 3.4.1. Discussion Point

GIT symptoms in COVID-19 patients are one of the primary and initial symptoms that are frequently seen in mild to moderate cases of COVID-19. The causes of GIT symptoms are as follows: viral overload, SARS-CoV-2 effect on ACE2 expression in GIT, and the cytokine storm. COVID-19 patients present with late symptoms or complications. Diarrhea or loose motion is the most frequent primary symptom in COVID-19 patients. However, late complications that occur in severe infection or critical cases may include GIT symptoms such as liver injury, pancreatic injury, and GIT bleeding. GIT endoscope is safe for use in GIT bleeding management. Epigastric pain and abdominal pain were considered as primary manifestations of COVID-19 and also as COVID-19-related GIT manifestations due to gastritis and abdominal colic.

### 3.5. Renal Manifestations

The most important renal manifestation of COVID-19 is AKI. Some studies have shown an increased risk of mortality in COVID-19 patients who have AKI to a greater extent than the other patients who have normal kidney function (88, 89). Haematuria and proteinuria were reported as renal complications of COVID-19 (90). Some authors have suggested the direct effect of SARS-CoV-2 on the kidney because ACE2 is expressed in the kidney tissue (91). The ACE2 receptor is found in renal tubules and may be affected by SARS-CoV-2 (92). The cytokine storm affects kidney function and may induce acute renal failure (89). Increased blood levels of creatinine and blood urea nitrogen (BUN) are considered to increase the risk of hospital death in COVID 19 patients in the ICU (90). A post-mortem study showed the histopathological feature of acute renal tubular necrosis in patients who died due to COVID-19 (78). The viral load may also play a role in the renal manifestations of COVID-19 (93). Some studies have shown renal function deterioration in patients with severe pneumonia (94). Patients with chronic diseases, such as DM and chronic kidney disease, may have a higher risk of kidney function deterioration with COVID-19 along with a high mortality rate (95). Hyperkalaemia may occur in hospitalized patients (96). Refer to Table 4.

**Table 4:** Acute renal manifestations in COVID 19.

**Renal manifestation**	**References**	**Number of total patients**	**The affected patients**	**The incidence**
**Haematuria**	**Pei, Guangchang et al. “Renal Involvement and Early Prognosis in Patients with COVID-19 Pneumonia.” Journal of the American Society of Nephrology : JASN vol. 31,6 (2020): 1157-1165. doi: ** 10.1681/ASN.2020030276	**333**	**Haematuria 139/333**	**(41.7%)**
**proteinuria**			**Proteinuria 219/333**	**65.8%**
**Acute kidney injury**			**35/333**	**10.5%**
**Acute tubular necrosis[Arteriosclerosis ]**	**Su, Hua et al. “Renal histopathological analysis of 26 postmortem findings of patients with COVID-19 in China.” Kidney international vol. 98,1 (2020): 219-227. doi: ** 10.1016/j.kint.2020.04.003	**26 postmortem findings of patients with COVID-19 in China**		
**Hyperkalemia**	**Malieckal, Deepa A et al. “Electrolyte abnormalities in patients hospitalized with COVID-19.” Clinical kidney journal vol. 14,6 1704-1707. 16 Mar. 2021, doi:** 10.1093/ckj/sfab060	**10348**	**638**	**6.6%**

#### 3.5.1. Discussion Point

Acute kidney injury is considered one of the COVID-19 manifestations in severe and critical cases. It may also occur as a complication of COVID-19 pneumonia. Viral overload and comorbidity may play a role in determining the severity of AKI in COVID-19. In high viral overload, the ACE2 receptor has a direct effect from SARS-CoV-2, leading to AKI, and a cytokine storm that results from high infection may lead to AKI. Comorbidities, such as diabetic mellitus and chronic kidney disease, are predisposing factors for AKI in COVID-19 patients or acute on top of chronic kidney disease in COVID-19 patients. Hyperkalaemia is a complication of acute renal failure or AKI. Proteinuria and haematuria are considered manifestations of AKI.

### 3.6. The Neurological Manifestations

The neurological manifestations of COVID-19 may be central or peripheral symptoms (97). The central nervous symptom manifestations may be headaches, dizziness, ischaemic stroke, intracranial hemorrhage, encephalitis, seizures, loss of smell (dysgeusia), or insomnia (98). Peripheral nervous manifestations may include neuropraxia, ophthalmoplegia, ataxia, loss of tendon reflex, Miller Fisher syndrome, and Guillain–Barre syndrome (99). Acute transverse myelitis with hypotonia has been reported in some patients with COVID-19 (100). The mechanism of neurological manifestations of COVID-19 may be because the SARS-CoV-2 exerts a direct effect on ACE2 receptors that are distributed in brain tissue (101). Some authors suggested that SARS-CoV-2 could be ascending by olfactory nerve axons to the brain and thalamus (101). The autopsy specimen showed brain edema and degeneration in the nerve ending by a post-mortem study in the dead body of COVID-19 (98). SARS-CoV 2 has a hypercoagulopathy effect, which can explain the ischaemic stroke incident in COVID-19 patients (102). Some studies reported high fever with a disturbed consciousness level in some of the COVID-19 patients, and cerebrospinal fluid analysis showed the presence of SARS-CoV-2. The patients were diagnosed with COVID-19 and viral encephalitis (103). Seizure disorders have been previously reported in hospitalized COVID-19 patients without any history of epilepsy. Retrospective studies of hospitalized patients reported headache and dizziness in COVID-19 patients (99, 104). Cerebral hemorrhage occurs in some COVID-19 patients in the ICU (105). Some retrospective studies reported one patient with cerebral venous thrombosis and another with cerebral hemorrhage (106). Insomnia was also reported in some COVID-19 patients even after negative PCR results (107). Early neurological symptoms of mild COVID-19 patients may involve loss of smell, headache, and dizziness. Some cases reported about Guillain–Barre syndrome in a patient with COVID-19 also reported Miller Fisher syndrome (108, 109). Some retrospective studies showed peripheral neurological symptoms in COVID-19 patients with ophthalmoplegia and polyneuritis (110). Ataxia has been reported in COVID-19 patients (111). Acute myelitis with hypotonia and loss of tendon reflex are also seen (112). Refer to Table 5.

**Table 5:** Neurological manifestations of COVID 19.

**Type of Neurological Disease**	**Manifestations**	**References**	**Number of total patients**	**The affected patients**	**The Incidence**
**Central nervous system**	**Headache**	**Mao, Ling et al. “Neurologic Manifestations of Hospitalized Patients With Coronavirus Disease 2019 in Wuhan, China.” JAMA neurology vol. 77,6 (2020): 683-690. doi: ** 10.1001/jamaneurol.2020.1127	**214**	**28**	**13.1%**
	**Dizziness**			**36**	**16.8%**
	**Seizures**			**2**	**2%**
	**Cerebral haemorrhage**	**Craen, Alexandra et al. “Novel Coronavirus Disease 2019 and Subarachnoid Hemorrhage: A Case Report.” Cureus vol. 12,4 e7846. 27 Apr. 2020, doi:** 10.7759/cureus.7846	**Case report**	**Case report**	
	**Acute cerebrovascular disease**	**Mao, Ling et al. “Neurologic Manifestations of Hospitalized Patients With Coronavirus Disease 2019 in Wuhan, China.” JAMA neurology vol. 77,6 (2020): 683-690. doi: ** 10.1001/jamaneurol.2020.1127	**214**	**6**	**2.8 %**
	**Ischemic stroke**	**Tan, Y. K., Goh, C., Leow, A., Tambyah, P. A., Ang, A., Yap, E. S., Tu, T. M., Sharma, V. K., Yeo, L., Chan, B., & Tan, B. (2020). COVID-19 and ischemic stroke: a systematic review and meta-summary of the literature. Journal of thrombosis and thrombolysis, 50(3), 587–595. doi: ** 10.1007/s11239-020-02228-y	**362**	**9**	**2.5%**
	**Insomnia**	**Kokou-Kpolou, Cyrille Kossigan et al. “Insomnia during COVID-19 pandemic and lockdown: Prevalence, severity, and associated risk factors in French population.” Psychiatry research vol. 290 (2020): 113128. doi: ** 10.1016/j.psychres.2020.113128	**556**		**18.2%**
		**Huang, Yeen, and Ning Zhao. “Generalized anxiety disorder, depressive symptoms and sleep quality during COVID-19 outbreak in China: a web-based cross-sectional survey.” Psychiatry research vol. 288 (2020): 112954. doi: ** 10.1016/j.psychres.2020.112954	**7236**	**1317**	**18.2%**
	**cerebral venous thrombosis**	**Garaci, Francesco et al. “Venous cerebral thrombosis in COVID-19 patient.” Journal of the neurological sciences vol. 414 (2020): 116871. doi: ** 10.1016/j.jns.2020.116871	**Case report**		
	**Ataxia**	**Mao, Ling et al. “Neurologic Manifestations of Hospitalized Patients With Coronavirus Disease 2019 in Wuhan, China.” JAMA neurology vol. 77,6 (2020): 683-690. doi: ** 10.1001/jamaneurol.2020.1127	**214**	**1**	**0.5%**
**Peripheral neurological manifestation**	**Gillian bare syndrome**	**Sedaghat Z, Karimi N. Guillain Barre syndrome associated with COVID-19 infection: A case report. J Clin Neurosci. 2020 Jun;76:233-235. doi: **10.1016/j.jocn.2020.04.062 **Epub 2020 Apr 15. PMID: 32312628; PMCID: PMC7158817**	**Case report**		
	**ophthalmoplegia and polyneuritis**	**Guidon AC, Amato AA. COVID-19 and neuromuscular disorders. Neurology. 2020; 94(22):959–69**	**Review**		
		**Mao, Ling et al. “Neurologic Manifestations of Hospitalized Patients With Coronavirus Disease 2019 in Wuhan, China.” JAMA neurology vol. 77,6 (2020): 683-690. doi: ** 10.1001/jamaneurol.2020.1127	**214**	**Loss Taste in 12 patients**	**5.6%**
				**Loss Smell in 11 patients**	**5.1%**
				**Vision Defect in 3 patients**	**1.4%**
				**Nerve pain in 5 patients**	**2.3%**
				**Skeletal muscle injury in 23 patients**	**10.7%**
	**Acute myelitis and Hypotonia**	**AlKetbi, Reem et al. “Acute myelitis as a neurological complication of Covid-19: A case report and MRI findings.” Radiology case reports vol. 15,9 1591-1595. 6 Jun. 2020, doi:** 10.1016/j.radcr.2020.06.001		**Case report**	

#### 3.6.1. Discussion Point

The neurological manifestations of COVID-19 may include primary symptoms such as headache, dizziness, or loss of smell, or late complications such as cerebral hemorrhage and seizures. Neurological disorders may occur as complications of vascular thromboembolic disorder (VTE), causing brain ischaemia and stroke. The mechanism of nervous system defects in COVID-19 patients is as follows: (1) The direct effect of SARS-CoV-2 on ACE2 receptors in the brain and nerve; some authors mention that SARS-CoV-2 can ascend from the olfactory nerve from the nasal cavity to the brain thalamus, (2) COVID-19 hypoxia may have an effect on brain tissue and could lead to brain oedema, (3) the cytokine storm may have an effect on the central and peripheral nervous systems, (4) the VTE effect of COVID-19 may lead to brain ischaemia and stroke. Many psychosis cases were reported in COVID-19 patients. Stroke and cerebral hemorrhage were reported in COVID-19 patients and acute myelitis with hypotonia in a patient with COVID-19. Gillian–Barre syndrome was also reported in COVID-19 patients. The effect of COVID-19 on the peripheral nervous system may be determined by the degree of infection as a viral load and the severity of the case that can cause nervous system complications, as well as the effect of the virus on the nervous tissue or the complication of hypoxia and thromboembolic effect that causes nerve ischaemia. Genetic factors and comorbidities may have a role in the onset of nervous system manifestations.

### 3.7. Psychiatric Manifestations

Coronavirus disease-2019 has two categories of psychological manifestations (113). The first is psychiatric symptoms as a result of isolation and quarantine, and this type is frequent and showed depression, anxiety, sleeping disorders, eating disorders, somatizations, and phobias. The second category is the psychiatric disorder as a result of the effect of COVID-19 itself (SARS-CoV-2 in the brain tissue). This type may be rare but was reported in a case series study for some patients with COVID-19 who developed psychosis in Spain (114). One observational study conducted among patients in isolation reported depression and suicidal ideation in some of them. Isolated and quarantine people need psychometric support during the isolation period and after isolation follow-up (115, 116). Refer to Table 6.

**Table 6:** Psycahtric manifestations of COVID 19.

**Manifestation**	**References**	**study sample**	**Affected patients / people**	**Incidence**
**Psycahtric symptoms as result of isolation and quarantineSuch as :depression**,	**Zhang, Jie et al. “The differential psychological distress of populations affected by the COVID-19 pandemic.” Brain, behavior, and immunity vol. 87 (2020): 49-50. doi: ** 10.1016/j.bbi.2020.04.031	**57**	**Depression Mild 18** **Moderate 7** **Severe 11**	**31.6%** **12.3%** **19.3%**
**anxiety**,			**Anxiety : Mild 14** **Moderate 4** **Severe 8**	**36.8 %26.7%32.0 %**
**sleeping disorders**,	**Huang, Yeen, and Ning Zhao. “Generalized anxiety disorder, depressive symptoms and sleep quality during COVID-19 outbreak in China: a web-based cross-sectional survey.” Psychiatry research vol. 288 (2020): 112954. doi: ** 10.1016/j.psychres.2020.112954	**7236**	**1317**	**18.2 %**
**eating disorders**,	**Fernández-Aranda, Fernando et al. “COVID-19 and implications for eating disorders.” European eating disorders review : the journal of the Eating Disorders Association vol. 28,3 (2020): 239-245. doi: ** 10.1002/erv.2738	**32**	**About 12**	**37.5%**
**Psychosis after COVID 19 infection**	**Rentero, D., Juanes, A., Losada, C. P., Álvarez, S., Parra, A., Santana, V., Mart**í**, I., & Urricelqui, J. (2020). New-onset psychosis in COVID-19 pandemic: a case series in Madrid. Psychiatry research, 290, 113097. doi: **10.1016/j.psychres.2020.113097	**a case series**		
**suicidal ideation**	**Czeisler, Mark É et al. “Mental Health, Substance Use, and Suicidal Ideation During the COVID-19 Pandemic - United States, June 24-30, 2020.” MMWR. Morbidity and mortality weekly report vol. 69,32 1049-1057. 14 Aug. 2020, doi:** 10.15585/mmwr.mm6932a1	**5,470**	**About 585**	**10.7 %**

#### 3.7.1. Discussion Point

The psychiatric manifestation of COVID-19 may be the result of the direct effect of SARS-CoV-2 on ACE2 receptors in the brain, which may manifest as psychosis, lack of sleep, insomnia, and anxiety. The presentation of psychiatric manifestations of COVID-19 as a primary symptom may infrequently be reported and seen in practice. The other type of psychiatric manifestation of COVID-19 resulted from secondary causes, such as isolation- and quarantine-related depression. Depression was reported in people both in quarantine and in some health care providers who manage COVID-19 patients. Suicidal ideation was reported in patients in quarantine and isolation. Therefore, psychiatric support and follow-up may be required for people in isolation even in health care providers for COVID-19.

### 3.8. Skin and Dermatological Manifestations of COVID-19

Some authors mention the basal layer of the skin as the location of ACE2 receptors in the human body (117). Two pathophysiological mechanisms are underlying the cutaneous manifestations of COVID-19. The first is the direct effect of SARS-CoV-2 on ACE2 receptors in the basal layer of the skin, and the second mechanism may be complicated by the effects of drugs that are used to treat COVID-19, such as skin side effects due to azithromycin.

The cutaneous manifestations of COVID-19 may appear as erythema maculopapular redness, skin rash, and erythema. Some of the quarantine patients experienced skin erythema after a few days of quarantine. One study showed that COVID-19 patients presented with skin rash and erythema. Skin manifestations of COVID-19 may present before respiratory symptoms (118–123). Refer to Table 7.

**Table 7:** Skin manifestations of COVID 19.

**Manifestations**	**References**	**Sample size**	**Affected patients**	**Incidence**
**Macular popular rash, skin rash and Erythema**	**Sachdeva M, Gianotti R, Shah M, Bradanini L, Tosi D, Veraldi S, Ziv M, Leshem E, Dodiuk-Gad RP, 2020. Cutaneous manifestations of COVID-19: report of three cases and a review of literature. J Dermatol Sci 98: 75–81**	**3 case report**		**I agree with the result I have seen COVID 19 with cutaneous manifestations in my practice in field hospital FHOIA, HMC, Qatar**.
	**Joob B, Wiwanitkit V, 2020. COVID-19 can present with a rash and be mistaken for dengue. J Am Acad Dermatol 82: e177**.	**Case report**		
	**Recalcati S. Cutaneous manifestations in COVID-19: a first perspective. J Eur Acad Dermatol Venereol. 2020;34(5):e212–3**.	**88**	**18**	**20.4%**
	**Tammaro A, Adebanjo GAR, Parisella FR, Pezzuto A, Rello J. Cutaneous manifestations in COVID-19: the experiences of Barcelona and Rome. J Eur Acad Dermatol Venereol. 2020;34(7):e306–7**	**130**	**2**	**1.5%**

#### 3.8.1. Discussion Point

Skin manifestations of COVID-19 are reported as primary manifestations of COVID-19, also reported as late manifestations in patients in isolation and quarantine. The pathophysiological process may be the primary effect of SARS-CoV-2 on ACE2 in the basal layer of the skin, or secondary to the cytokine storm effect, or drug reaction used in COVID-19 management. Genetic factors may have a role in the appearance of the dermatological feature of COVID-19. The dermatological manifestations of COVID-19 are reported in mild and moderate cases of COVID-19.

### 3.9. Ocular Manifestations

The ocular manifestations of COVID-19 may occur through the direct effect of SARS–CoV-2, which is transmitted by droplet infection to the cornea and conjunctiva. The virus present in the droplets binds with ACE2 receptors located in the conjunctiva and cornea. SARS-CoV-2 may reach the eyes through systematic circulation according to the viral load. Some case reports showed ocular manifestation in COVID-19 as a burning eye sensation with redness. In addition, foreign body sensation and conjunctivitis were reported in COVID-19 cases. Some studies have recommended the use of Goggles to protect the eyes as a site of COVID-19 transmission. Some studies have mentioned conjunctivitis as an early symptom of COVID-19 (26, 27, 124–128). Refer to Table 8.

**Table 8:** Ocular manifestations of COVID 19.

**Manifestations**	**Reference**	**The sample size**	**Affected patients**	**Incidence**
	**Zhang X, Chen X, Chen L, Deng C, Zou X, Liu W, Yu H, Chen B, Sun X, 2020. The evidence of SARS-CoV-2 infection on ocular surface. Ocul Surf 18: 360–362**.	**72 confirmed COVID 19 by laboratory diagnosis**	**2 of 72**	**About 2.78%**
**Eye redness, conjunctivitis, foreign body sensation and burning eye sensation**	**Chen, Liwen et al. “Ocular manifestations and clinical characteristics of 535 cases of COVID-19 in Wuhan, China: a cross-sectional study.” Acta ophthalmologica vol. 98,8 (2020): e951-e959. doi: ** 10.1111/aos.14472	**535**	**27** **4 patient has conjunctivitis as initial symptoms**	**5 %** **0.7% of total patient and 14.8 % of patient with ocular symptoms**
	**Wu P, Duan F, Luo C, Liu Q, Qu X, Liang L, Wu K, 2020. Characteristics of ocular findings of patients with coronavirus disease 2019 (COVID-19) in hubei province, China. JAMA Ophthalmol 138: 575–578**.	**38**	**12**	**31.5%**

#### 3.9.1. Discussion Point

There are two ways for SARS–CoV-2 to reach the eye tissue. In the first method, droplet infection reaches the eye tissue by direct contact, and in the second method, it reaches through blood circulation, which occurs according to the viral overload. The cytokine storm may have an effect on eye manifestations. Eye manifestations are reported as primary manifestations of COVID-19 and may occur as late manifestations.

### 3.10. Endocrinology Manifestations of COVID-19

Some observational studies have shown abnormalities in thyroid function in COVID-19 patients. COVID-19 is a cause of ketosis in non-diabetes patients and may also create a high risk for diabetic keto acidosis (DKA) in diabetes patients. One study reported ketosis in a COVID-19 patient without any hyperglycaemia, vomiting, or fever. In addition, the pancreatic injury was reported in 19 cases, and amylase and lipase levels were elevated. The mechanism of endocrine manifestations may be because some endocrine glands express ACE2, such as thyroid and pancreas, or may be due to the hyperimmune feature of the cytokine storm. Adrenal insufficiency may occur as secondary adrenal insufficiency in COVID-19 patients as a result of pituitary hypofunction. Primary adrenal insufficiency is reported as a result of a thrombotic cause and it is a sign of worsening in ARDS. The unexplained body aches in COVID-19 patients may result from a defect in the hypothalamus–pituitary–adrenal axis, which leads to defective production of ACTH, causing secondary adrenal insufficiency (131–137) (129–135). Refer to Table 9.

**Table 9:** Endocrine manifestation of COVID 19.

**Manifestations**	**Reference**	**Sample size**	**Affected patients**	**Incidence**
**Ketosis**	**Li, Juyi et al. “COVID-19 infection may cause ketosis and ketoacidosis.” Diabetes, obesity & metabolism vol. 22,10 (2020): 1935-1941. doi: ** 10.1111/dom.14057	**658**	**42 [no obvious fever or diarrhoea]**	**About 6.4%**
**DKA**	**Li, Juyi et al. “COVID-19 infection may cause ketosis and ketoacidosis.” Diabetes, obesity & metabolism vol. 22,10 (2020): 1935-1941. doi: ** 10.1111/dom.14057	**658 all the patients**	**3 patients had DKA** **42 had ketosis**	**0.455 % From total patient** **6.4 % out of 658 patients had ketosis at admission time, they have no obvious fever or diarrhoea**
	**Gentile, Sandro et al. “COVID-19, ketoacidosis and new-onset diabetes: Are there possible cause and effect relationships among them?.” Diabetes, obesity & metabolism vol. 22,12 (2020): 2507-2508. doi: ** 10.1111/dom.14170	**Review**		
**New onset of hyperglycaemia**	**Ghosh, Amerta, and Anoop Misra. “Marked hyperglycemia and ketosis in a non-obese patient with new onset diabetes and very mild COVID-19 symptoms: A case report.” Diabetes & metabolic syndrome vol. 15,1 (2021): 213-214. doi: ** 10.1016/j.dsx.2020.12.036	**Case report**		**I Agree with this case result,Seen frequently in my practice in Field hospital FHOIA, HMC, Qatar**
	**Bode, Bruce et al. “Glycemic Characteristics and Clinical Outcomes of COVID-19 Patients Hospitalized in the United States.” Journal of diabetes science and technology vol. 14,4 (2020): 813-821. doi: ** 10.1177/1932296820924469	**1122**	**257**	**About 22.9% of total patient**
**Abnormal thyroid function**	**Chen, Min et al. “Thyroid Function Analysis in 50 Patients with COVID-19: A Retrospective Study.” Thyroid : official journal of the American Thyroid Association, 10.1089/thy.2020.0363. 10 Jul. 2020, doi:** 10.1089/thy.2020.0363	**50**	**32**	**64%**
**Adrenal insufficiency**	**Almeida, Madson Q, and Berenice B Mendonca. “Adrenal Insufficiency and Glucocorticoid Use During the COVID-19 Pandemic.” Clinics (Sao Paulo, Brazil) vol. 75 e2022. 12 Jun. 2020, doi:** 10.6061/clinics/2020/e2022	**review**		
	**Heidarpour, M., Vakhshoori, M., Abbasi, S. et al. Adrenal insufficiency in coronavirus disease 2019: a case report. J Med Case Reports 14, 134 (2020). doi: ** 10.1186/s13256-020-02461-2	**Case report**		
	**Hashim M, Athar S, Gaba WHNew onset adrenal insufficiency in a patient with COVID-19BMJ Case Reports CP 2021;14:e237690**.	**case report**		
**defect in hypothalamus - pituitary axis**	**Alzahrani, Ali S et al. “The Impact of COVID-19 Viral Infection on the Hypothalamic-Pituitary-Adrenal Axis.” Endocrine practice : official journal of the American College of Endocrinology and the American Association of Clinical Endocrinologists vol. 27,2 (2021): 83-89. doi: ** 10.1016/j.eprac.2020.10.014	**28**	**ACTH Level** **<** **10 in [7] patients** **Level** **<** **20 in [17] patients** **Level** **<** **30 in [23] patients**	**25%** **60.7%** **82.1 %**
			**Cortisol level** **Level** **<** **100 in [8] patients Level** **<** **200 in [14] patients Level** **<** **300 in [18] patients**	**28.6%** **50 %** **64.3%**
**Pancreatic injury**	**Bansal, Priya et al. “Pancreatic Injury in COVID-19 Patients.” The Journal of the Association of Physicians of India vol. 68,12 (2020): 58-60**.	**42**	**14 patients had increase in amylase level**	**33%**
		**29**	**7 patient had increase in lipase level**	**24.1%**

#### 3.10.1. Discussion Point

The endocrine manifestations of COVID-19 may occur as late manifestations. It has been reported in hospitalized patients. In addition, it may occur as an initial manifestation, such as DKA in COVID-19 patients with diabetic mellitus. COVID-19 is one of the causes of ketosis and DKA in diabetes patients. ACE2 expression is found in many endocrine glands such as the thalamus, thyroid, pituitary, and pancreas, which explain the effect of COVID-19 on the endocrine system. The cytokine storm leads to multiorgan failure and plays a role in endocrine dysfunction. Blood tests for endocrine function may require monitoring of endocrine function in hospitalized COVID-19 patients.

### 3.11. Testosterone Level (Male Reproductive System)

Some studies have reported that testosterone (T) levels decreased in COVID-19 patients, while the luteinizing hormone (LH) levels increased. Therefore, the T: LH ratio may decrease in COVID-19 patients (136). The authors mention the testes as one of the ACE2 receptor sites in the body; however, one study reported that no RNA of SARS-CoV-2 was found in testicular tissue biopsy of COVID-19 patients, but the decrease in the T level suggests Leydig cell damage in the testes (137, 138). However, one study suggested that high levels of cytokines in COVID-19 patients may lead to defects in the function of testes and spermatogonia (139). Refer to Table 10.

**Table 10:** The defect in testosterone in COVID 19.

**The Defect**	**Reference**	**The sample size**	**The affected patients**	**Incidence**
	**Okçelik, Sezgin. “COVID-19 pneumonia causes lower testosterone levels.” Andrologia, e13909. 19 Nov. 2020, doi:** 10.1111/and.13909	**24**	**9 patients have low testosterone**	**37.5%**
			**7 patients have high LH**	**About 29%**
**Decrease Testosterone [T] levelIncrease luteinizing hormone LHT: LH may decrease in COVID 19 patient**	**Wang, Zhengpin, and Xiaojiang Xu. “scRNA-seq Profiling of Human Testes Reveals the Presence of the ACE2 Receptor, A Target for SARS-CoV-2 Infection in Spermatogonia, Leydig and Sertoli Cells.” Cells vol. 9,4 920. 9 Apr. 2020, doi:** 10.3390/cells9040	**16,632 cells**	**ACE2 expression finding in testicular cells with variations**	**ACE2 represented as :1.4% in spermatogonia4.25% in Leydig and Sertoli cells**
	**Yang, Ming et al. “Pathological Findings in the Testes of COVID-19 Patients: Clinical Implications.” European urology focus vol. 6,5 (2020): 1124-1129. doi: ** 10.1016/j.euf.2020.05.009	**12**	**11**	**About 91 % have testicular injury**

#### 3.11.1. Discussion Point

Testosterone decline may be a complication of COVID-19. ACE2 expression is found in testicular tissue and may be affected by SARS-CoV-2. Cytokine storm has an effect on testicular function. Testosterone level follow-up is required for hospitalized male COVID-19 patients.

### 3.12. Pregnancy

ACE expression is found in the placenta and umbilical cord (140). Studies showed that neonates were infected with SARS-CoV-2. There is no confirmed information about the time of viral infection from the mother to the fetus, whether it occurs in the first, second, or third trimester (141). Disturbance in maternal–placental blood flow was reported in pregnant patients with COVID-19, and placental hypoxia was reported with systemic hypoxia as a result of COVID-19 in pregnant women (142, 143). Placental hypoxia and disturbance in placental blood flow may lead to a decrease in fetal growth, preterm birth, maternal death, and spontaneous abortion (144). Refer to Table 11.

**Table 11:** COVID 19 effect in pregnancy.

**COVID 19 effect in pregnancy**	**References**	**Sample size**	**Affected patients**	**Incidence**
	**Mahyuddin, Aniza P et al. “Mechanisms and evidence of vertical transmission of infections in pregnancy including SARS-CoV-2s.” Prenatal diagnosis vol. 40,13 (2020): 1655-1670. doi: ** 10.1002/pd.5765	**40 case series**		
	**Shanes, Elisheva D et al. “Placental Pathology in COVID-19.” American journal of clinical pathology vol. 154,1 (2020): 23-32. doi: ** 10.1093/ajcp/aqaa089	**15**	**12 cases had maternal vascular mal-perfusion**	**12/15**
**Vertical infectionDisturbance in maternal placenta blood flowPlacental hypoxia**	**Wenling, Yao et al. “Pregnancy and COVID-19: management and challenges.” Revista do Instituto de Medicina Tropical de Sao Paulo vol. 62 (2020): e62. doi: ** 10.1590/s1678-9946202062062	**Review**		
**decrease in foetal growth, preterm birth, maternal death and spontaneous abortion**	**Sheth, Sudip et al. “Outcomes in COVID-19 Positive Neonates and Possibility of Viral Vertical Transmission: A Narrative Review.” American journal of perinatology vol. 37,12 (2020): 1208-1216. doi: ** 10.1055/s-0040-1714719	**Review**		
	**Chi J, Gong W, Gao Q. Clinical characteristics and outcomes of pregnant women with COVID-19 and the risk of vertical transmission: a systematic review. Arch Gynecol Obstet. 2020 Dec 1:1–9. doi: **10.1007/s00404-020-05889-5 **Epub ahead of print. PMID: 33258995; PMCID: PMC7706177**.	**Systematic review**	**230**	**34.62 % had obstetrics complications**
				**59.05% displayed fever**
				**40.71% had lymphopenia**
				**5.19% received mechanical ventilation7 women were critical ill**
				**24.74% of new-borns were premature**
				**5 of new-borns had positive COVID PCR**
				**8 of new born had negative COVOD PCR and 3 of them had elevated IgM and IgG against SARS –COV2**

#### 3.12.1. Discussion Points

COVID-19 has an effect on pregnancy patients in the form of vertical transmission of the disease from the mother to the fetus through the umbilical cord. The effect of COVID-19 in pregnancy depended on the following factors: (1) direct effect of SARS-CoV-2 on ACE2 receptors on the umbilical cord and placenta, (2) effect of hypoxia on COVID-19 pneumonia, and (3) cytokine storm. Pregnant COVID-19 patients need observation and follow-up about fetal growth, placental blood flow, and maternal blood oxygen levels.

### 3.13. Coagulopathy Manifestations

Angiotensin-converting enzyme-2 has receptors on endothelial cells, indicating that SARS-CoV-2 has a direct effect on endothelial integrity that can induce VTE disease, which can cause deep vein thrombosis (DVT) and pulmonary embolism (PE) (145). The studies showed DVT and PE in hospitalized COVID-19 patients, especially in old and ICU patients (146). The D-dimer level increases in COVID-19 patients (147). Disseminated intermittent coagulopathy (DIC) is reported in many cases of hospitalized COVID-19 patients (148). Platelets could be decreased in COVID-19 (149). One study suggested that ACE2 receptors in platelets indicate the direct effect of SARS-CoV-2 on platelets, leading to platelet hyperactivity and increased thrombus formation (150). Refer to Table 12.

**Table 12:** Coagulopathy manifestation of COVID 19.

**Coagulopathy manifestations**	**References**	**Sample size**	**Affected patients**	**Incidence**
**Effect on endothelial integrity that can induced venous thrombo embolic disease VTE as DVT and PE**	**Nägele, Matthias P et al. “Endothelial dysfunction in COVID-19: Current findings and therapeutic implications.” Atherosclerosis vol. 314 (2020): 58-62. doi: ** 10.1016/j.atherosclerosis.2020.10.014	**Review**		
	**Middeldorp, Saskia et al. “Incidence of venous thromboembolism in hospitalized patients with COVID-19.” Journal of thrombosis and haemostasis : JTH vol. 18,8 (2020): 1995-2002. doi: ** 10.1111/jth.14888	**198**	**Venous thromboembolism in 39 patients**	**20%**
			**DVT in 26 patients**	**13 %**
			**Symptomatic VTE in 25 patients**	**About 13%**
**High D dimer**	**Li, Y., Zhao, K., Wei, H., Chen, W., Wang, W., Jia, L., Liu, Q., Zhang, J., Shan, T., Peng, Z., Liu, Y., & Yan, X. (2020). Dynamic relationship between D-dimer and COVID-19 severity. British journal of haematology, 190(1), e24–e27. doi: ** 10.1111/bjh.16811	**279**	**140 patients**	**123 patients had much elevation in D dimer with poor prognosisAnd 23 patients had improved**
**Low platelet**	**Zhao, Xiaofang et al. “Early decrease in blood platelet count is associated with poor prognosis in COVID-19 patients-indications for predictive, preventive, and personalized medical approach.” The EPMA journal, vol. 11,2 1-7. 14 May. 2020, doi:** 10.1007/s13167-020-00208-z	**532 all COVID 19 patients and all have low platelet on admission time**	**29 died [the platelet had no increase after the admission]**	**5.45 %**
**DIC**	**Seitz, Rainer, and Wolfgang Schramm. “DIC in COVID-19: Implications for prognosis and treatment?.” Journal of thrombosis and haemostasis : JTH vol. 18,7 (2020): 1798-1799. doi: ** 10.1111/jth.14878	**Letter to editor**		

#### 3.13.1. Discussion Points

Coagulopathy in COVID-19 leads to thromboembolic manifestations such as DVT, PE, and brain stroke. The endothelial cells has ACE2 receptors that may affected by SARS-COV2. So the blood vessels may lose their integrity, which leads to platelet aggregation and thrombus formation. DIC is a complication of COVID-19. The coagulopathy manifestations of COVID-19 may be a late manifestation of COVID-19; however, it could be reported as an initial manifestation or presentation.

### 3.14. Laboratory Manifestations of COVID-19

**Figure 4:**
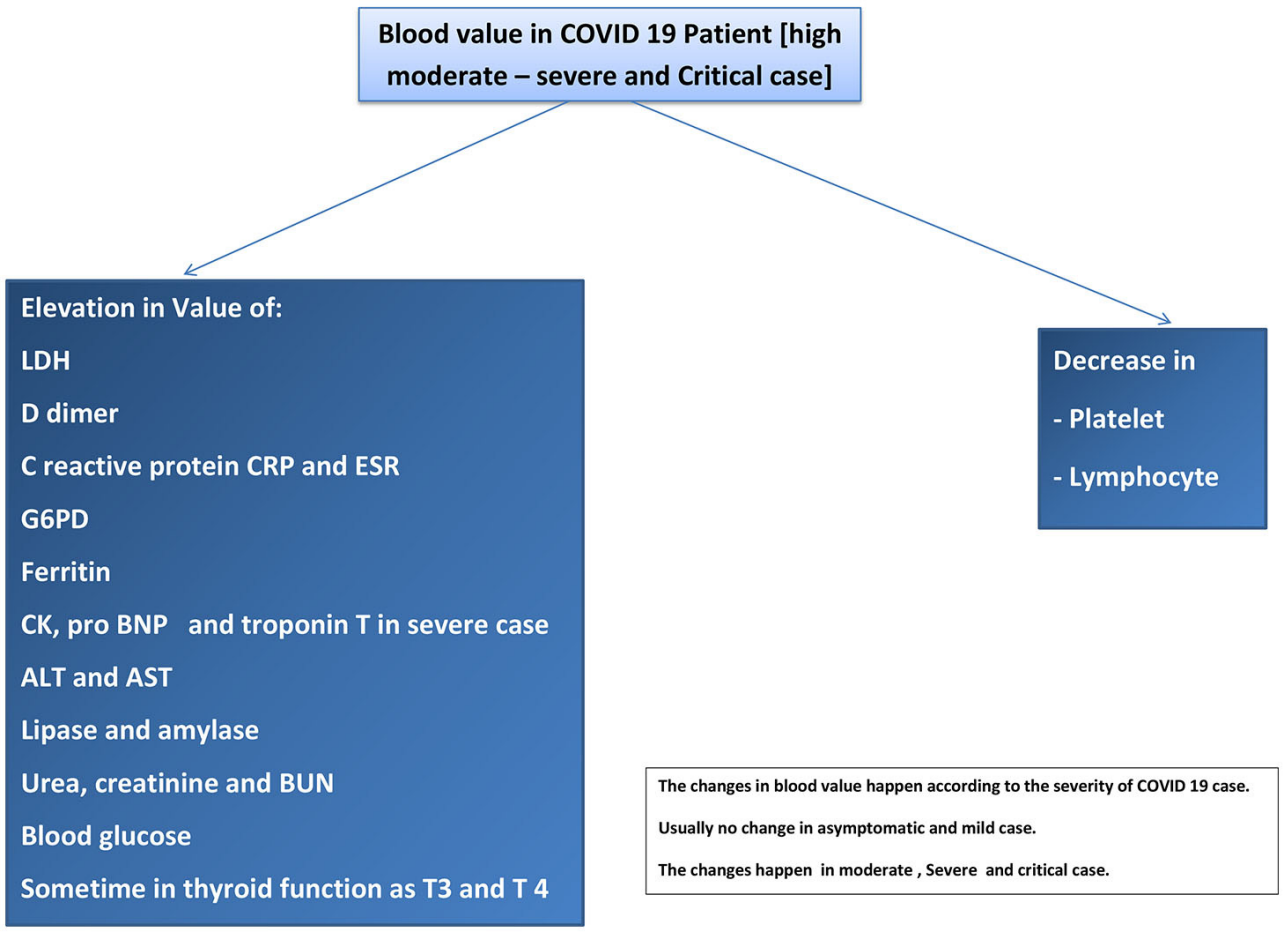
Blood values in COVID-19 patients (high severe and critical cases).

Many studies showed some changes in blood test results as abnormal values in COVID-19 patients. The studies recorded decreased lymphocyte and platelet counts and increased lactate dehydrogenase (LDH), D-dimer, prothrombin time PT, C-reactive protein (CRP), G6PD, and ferritin (151). Many studies have suggested the severity of the disease according to the blood value. For example, COVID-19 patients in the ICU have a low lymphocyte count (lymphopenia), high LDH, high D-dimer, and high prothrombin time (152). Another study suggested that patients need critical care if they have high D-dimer and CRP levels or thrombocytopenia and lymphopenia (39). The risk of ARDS is high in COVID-19 patients who have high D-dimer and low lymphocyte levels (153). The mortality rate increased with the patients who had high D-dimer levels. CK was elevated in COVID-19 patients and in that case rhabdomyolysis was reported in a COVID-19 patient (154). Liver injuries were reported in some COVID-19 patients with elevated ALT and AST levels. AKI that occurs in COVID-19 patient is associated with an elevation in urea, creatinine, and BUN (155). Refer to Figure 4.

#### 3.14.1. Discussion Points

In general, blood testing is a diagnostic and monitoring method. The laboratory features in ICU and hospitalized COVID-19 patients may have increased CRP, ferritin, creatinine, urea, LDH, liver enzymes, lipase, D-dimer, and PT. Blood tests may show a decrease in platelet and lymphocyte counts. Almost all changes in laboratory results occur as late manifestations or complications in moderate–severe cases of COVID-19.

### 3.15. Musculoskeletal Manifestations

Generalized bone ache and muscle pain were reported in COVID-19 patients (156). Rhabdomyolysis was reported in a COVID-19 patient with high CK and AKI (157). There are two mechanisms for muscle and bone aches in COVID-19 (161). The first is the direct effect of SARS–CoV-2 on the ACE 2 receptors in the muscle and cortex of the bone (158). The second mechanism is the inflammatory reaction of cytokine storm (164 and 161). Autoimmune myositis was reported in a COVID-19 patient (159). Generalized myopathy with muscle loss and weakness was reported in COVID-19 patients (110). Refer to Table 13.

**Table 13:** Musculoskeletal manifestations of COVID 19.

**Manifestation**	**References**
**Bone ache and muscle pain**	**Disser, Nathaniel P et al. “Musculoskeletal Consequences of COVID-19.” The Journal of bone and joint surgery. American volume vol. 102,14 (2020): 1197-1204. doi: **10.2106/JBJS.20.00847 **CDC. Symptoms of Coronavirus. CDC. Available at doi: **https://www.cdc.gov/coronavirus/2019-ncov/symptoms-testing/symptoms.html**. May 13, 2020; Accessed: June 26, 2020**.
**Rhabdomyolysis and increase in CK level**	**Paliwal, V. K., Garg, R. K., Gupta, A., & Tejan, N. (2020). Neuromuscular presentations in patients with COVID-19. Neurological sciences : official journal of the Italian Neurological Society and of the Italian Society of Clinical Neurophysiology, 41(11), 3039–3056. doi: ** 10.1007/s10072-020-04708-8
**Myositis**	**Beydon, Maxime et al. “Myositis as a manifestation of SARS-CoV-2.” Annals of the rheumatic diseases, annrheumdis-2020-217573. 23 Apr. 2020, doi:** 10.1136/annrheumdis-2020-217573
**Myopathy**	**Guidon, Amanda C, and Anthony A Amato. “COVID-19 and neuromuscular disorders.” Neurology vol. 94,22 (2020): 959-969. doi: ** 10.1212/WNL.0000000000009566

#### 3.15.1. Discussion Point

Coronavirus disease-140 presented with severe musculoskeletal pain in some people. The musculoskeletal manifestations may present as primary or initial symptoms or may present as late manifestations or complications such as myositis or myopathy. Rhabdomyolysis was reported in a COVID-19 patient that led to AKI.

### 3.16. Initial Manifestations (Symptoms) of COVID-19

The COVID-19 cases were divided into asymptomatic, mild, moderate, severe, and critical cases, according to the degree of infection and severity of the symptoms. Refer to Figures 1, 2.

The initial manifestations of COVID-19 vary. In the mild cases, the common symptoms were fever, cough, fatigue, musculoskeletal pain, headache, vomiting, loose motion, sore throat, cough, palpation, epigastric pain, and abdominal pain. COVID-19 coughs were reported to be an almost dry cough or sputum cough. In severe cases, the initial manifestations may be considered as high-risk symptoms such as respiratory failure, shock, respiratory distress, arrhythmia, AKI, severe bacterial infection may be secondary infection, sepsis, septic shock, and GIT bleeding. Peripheral neuropathy was reported as a primary manifestation of myopathy. A patient with a moderate case may present with shortness of breath (SOB) and bilateral pneumonia (160).

Usually, mild symptoms of COVID-19 are present in healthy young patients without any comorbidities. Severe symptoms may be present in patients with comorbidities or old age. Most at-risk patients who are exposed to COVID-19 may be old, pregnant, diabetic, and hypertensive, and have hepatic failure or related conditions, ischaemic heart disease, and chronic kidney disease (160).

Mild symptoms with extra pulmonary manifestations need health care providers with good clinical sense to diagnose COVID-19 (160, 161).

Some COVID-19 patients may be asymptomatic and may continue to be asymptomatic until cure. Some of them present with severe symptoms. Some of the authors have used the term “pre-symptomatic phase” for some patients who were first asymptomatic but then presented with symptoms of COVID-19. For example, many patients presented to the emergency department with an SOB, with low oxygen saturation, and with no history of cough or respiratory symptoms before and after the chest X-ray showed bilateral pulmonary infiltration in COVID-19 pneumonia. The incubation period for SARS-CoV-2 is 14 days, as suggested in some studies, and some studies report 11.5 days; we refer to the period before the onset of symptoms as the asymptomatic phase or the pre-symptomatic phase (161, 162).

The critical case of COVID-19 presented with ARDS or multiple organ failure. Some of the critical cases started as mild symptoms then worsened, and some of them were asymptomatic and experienced ARDS or multiple organ failure as sudden attacks (160, 162).

One of the controversies is COVID-19 reinfection. Some studies have reported immunity toward COVID-19 after infection recovery. Some of the patients had no immunoglobulin IgM or IgG after recovery from infection, which may support the theory of reinfection of COVID-19. However, no case reported ARDS as a result of reinfection of COVID-19. Viral RNA may continue after the symptoms resolve during the recovery, but this does not mean that the patient still has a viral infection (161–163). Refer to Table 14.

**Table 14:** COVID 19 manifestations in paediatric.

**Manifestations**	**References**
**Fever, cough, shortness in breath, loose motion, abdominal pain, muscle ache, nasal flue, vomiting, nasal congestion, skin Rash, conjunctivitis, sore throat and loss of sense of taste and smell**	- **CDC COVID-19 Response Team. Coronavirus disease 2019 in children - United States, February 12-April 2, 2020. MMWR Morb Mortal Wkly Rep. 2020 Apr 10. 69 (14):422-6. [Medline]**. - **Lu X, Zhang L, Du H, et al. SARS-CoV-2 infection in children. N Engl J Med. 2020 Apr 23. 382 (17):1663-65**. - **Castagnoli R, Votto M, Licari A, et al. Severe acute respiratory syndrome coronavirus 2 (SARS-CoV-2) infection in children and adolescents: a systematic review. JAMA Pediatr. 2020 Apr 22**. - **Foust AM, Phillips GS, Chu WC, et al. International expert consensus statement on chest imaging in pediatric COVID-19 patient management: imaging findings, imaging study reporting and imaging study recommendations. Radiology: Cardiothoracic Imaging. 2020 Apr 23. 2**

### 3.17. COVID-19 Manifestations and Diagnosis

The COVID-19 manifestation can help diagnose COVID-19 patients by Suspicion. For example, the Infectious Disease Society of America recommends some symptoms for COVID-19 tests such as critical ill patient, unexplained pneumonia or respiratory failure, fever, lower respiratory tract symptoms, contact with a positive case of COVID-19 within 14 days, recent travel during 14 days, immunosuppressed patient with a respiratory infection or recent admission to the hospital, and health care worker with fever or respiratory tract infection. However, there are other symptoms of COVID-19 such as headache, loose motion and loss of smell, recent insomnia, palpitation, abdominal pain, epigastric pain, recent arrhythmia, recent brain injury (stroke or hemorrhage) in any age group, signs of recent peripheral neuropathy, and AKI, in addition, the unexplained acute liver or pancreatic injury, recent thyroid function, disturbance, recent abortion, preterm delivery, or sudden fetal death in pregnant woman. We have to consider a new onset of hypertension, especially in young patients, without a family history and new-onset diabetes mellitus as probably symptoms of COVID19 infection. The principle is when the physician suspects COVID-19 manifestations as either pulmonary or extrapulmonary, COVID-19, the investigations are required to rule out COVID-19 infection. May be in the future the COVID-19 PCR or COVID-19 rapid antigen test will be routine investigations in some communities to rule out COVID-19 in unexplained symptoms or disease (31, 164).

Coronavirus disease investigations may involve laboratory or radiological investigations. Laboratory investigations include diagnostic studies such as the detection of SARS-CoV-2 RNA, antigen, or antibodies. Other laboratory tests may include leukopenia, leucocytosis, lymphopenia, increased LDH levels, high ferritin levels, unexplained ketosis, and high D-dimer levels (31).

The CT scan is a diagnostic tool for COVID-19, which reveals a bilateral ground-glass appearance that discovers silent pneumonia or the cause of silent hypoxia in many patients with COVID-19. The common feature of COVID-19 on a CT scan is the bilateral ground appearance; however, COVID-19 features may appear on CT as follows: bilateral peripheral patches, bilateral reticular opacity, bilateral congestion or vascular thickness, unfrequented pleural effusion (reported), and lymphadenopathy. However, the American College of Radiology recommends avoiding the use of CT as a screening tool to diagnose COVID 19, which should be used only for hospitalized patients. Other studies have reported the effectiveness of CT scans in diagnosing asymptomatic COVID-19 patients (165, 166).

An X-ray can be used to diagnose COVID-19 patients, as the X-ray findings in COVID-19 pneumonia are bilateral infiltration, patches, or glass-ground appearance (167).

### 3.18. Radiological Manifestations of COVID-19

We can explain the appearance of COVID-19 pneumonia in CT scans, such as multiple patches in the early phase, multiple ground-glass appearance in the moderate-to-severe case, and massive pulmonary consolidation in the severe case. As for X-ray findings, ground-glass appearance is the most common feature, followed by bilateral infiltration and multiple consolidations. Pleural effusion is infrequent, but has been reported (167, 168). Refer to Table 15.

**Table 15:** Radiological manifestations for pulmonary COVID 19.

**The Radiological Manifestation**	**The References**
**The X ray feature : the ground glass appearance is commonest, bilateral infiltration, multiple consolidations and Pleural effusion is infrequent but reported**	**- ACR. ACR Recommendations for the use of Chest Radiography and Computed Tomography (CT) for Suspected COVID-19 Infection. American College of Radiology. Available at doi: **https://www.acr.org/Advocacy-and-Economics/ACR-Position-Statements/Recommendations-for-Chest-Radiography-and-CT-for-Suspected-COVID19-Infection**. March 22, 2020; Accessed: April 2, 2020**.
**CT Feature : multiple patches in early phase, multiple ground glass appearance in moderate – sever case and massive pulmonary consolidation in the sever case**	**Wang Y, Liu Y, Liu L, Wang X, Luo N, Ling L. Clinical outcome of 55 asymptomatic cases at the time of hospital admission infected with SARS-Coronavirus-2 in Shenzhen, China. J Infect Dis. 2020 Mar 17**.
	**Bogoch, I. I., Watts, A., Thomas-Bachli, A., Huber, C., Kraemer, M., & Khan, K. (2020). Pneumonia of unknown aetiology in Wuhan, China: potential for international spread via commercial air travel. Journal of travel medicine, 27(2), taaa008. doi: ** 10.1093/jtm/taaa008
	**Li M, Lei P, Zeng B, Li Z, Yu P, Fan B, et al. Coronavirus Disease (COVID-19): Spectrum of CT Findings and Temporal Progression of the Disease. Acad Radiol. 2020 Mar 20**.

### 3.19. COVID-19 Manifestations in the Pediatric Patients

The most common symptoms of COVID-19 in children are fever, cough, SOB, loose motion, abdominal pain, muscle ache, nasal flu, vomiting, nasal congestion, skin rash, conjunctivitis, sore throat, and loss of sense of taste and smell. X-ray radiography showed bilateral ground-glass appearance, bilateral patches, and consolidation (169–172). Refer to Table 16.

**Table 16:** The initial manifestations [symptoms] of COVID 19.

**The Manifestations**	**References**
**Mild symptoms :Fever, cough, Fatigue, musculoskeletal pain, Headache, vomiting, loose motion, sore throat, cough, palpation, epigastric pain and abdominal pain**	**- CDC. Symptoms of Coronavirus. CDC. Available at doi: **https://www.cdc.gov/coronavirus/2019-ncov/symptoms-testing/symptoms.html**. May 13, 2020; Accessed: June 26, 2020**.
**The moderate case may present as SOB and bilateral pneumonia**.	**CDC. Coronavirus Disease 2019 (COVID-19): Evaluating and Testing PUI. Centers for Disease Control and Prevention. Available at doi: **https://www.cdc.gov/coronavirus/2019-ncov/hcp/clinical-criteria.html**. May 3, 2020; Accessed: June 9, 2020**.
**Sever symptoms :respiratory failure, shock, respiratory distress, arrhythmia, Acute kidney injury AKI, sever bacterial infection may as secondary infection, sepsis, septic shock, GIT bleeding**	**CDC. Interim Clinical Guidance for Management of Patients with Confirmed Coronavirus Disease (COVID-19). CDC. Available at doi: ** https://www.cdc.gov/coronavirus/2019-ncov/hcp/clinical-guidance-management-patients.html **. June 2, 2020; Accessed: June 9, 2020**
**Critical case :as acute respiratory distress syndrome or multiple organs failure**	**Gousseff, Marie et al. “Clinical recurrences of COVID-19 symptoms after recovery: Viral relapse, reinfection or inflammatory rebound?.” The Journal of infection vol. 81,5 (2020): 816-846. doi: ** 10.1016/j.jinf.2020.06.073

### 3.20. Role of Genes in COVID-19 Manifestations

Some authors believe in the role of the genes in determining the COVID-19 manifestations and the severity of the manifestations. The principle is that the ACE2 expression gene *G8790A* (rs2285666) has multiple polymorphisms (173). This means that it has different genotypes, such as A/A genotype, G/G genotype, and C/T genotype (174). The authors believe that the gene has different genotypes according to ethnicity. Different genotypes mean different RNA sequences and may differ in the susceptibility to SARS-CoV-2, differ in manifestations, and severity of the manifestations (175). Some Italian authors believe that the ACE2 genotype C/T is the most common in Italian people and is more susceptible to SARS-COV-2 than other genotypes in other ethnicities worldwide. The different susceptibility to SARS-CoV-2 means that some ethnicities are more affected by SARS-CoV-2 than others. Higher susceptibility means more severity and more critical manifestations, while low susceptibility means less severity with mild manifestations. Some researchers believe that some genotypes may confer resistance to SARS-CoV-2, which may explain asymptomatic patients (176).

On other hand, Some of the studies report that people with blood group A may have more susceptibility to COVID-19 in the severe form and manifestations, and those with blood group O may have low susceptibility and more resistance to COVID-19 (177, 178).

### 3.21. COVID-19 Manifestation and Management

COVID-19 patients were managed according to severity, the intensity of the manifestations, and type of manifestation, i.e., pulmonary and extrapulmonary. The asymptomatic case can be managed by isolation and quarantine, with a full observation of any symptoms that may be discovered by the patient. The mild case of COVID-19 may require quarantine with symptomatic treatment and close observation (179). The severe and moderate cases may require hospitalization, images (chest X-ray, CT scan according to the patient status), blood investigations, symptomatic treatment, and other types of management, specific to COVID-19, such as remdesvir, bamlanivimab, convalescent plasma, and baricitinib in children (2 years and above). For pulmonary manifestations, oxygen supplementation and mechanical ventilation are required according to the severity of the case. Some research and studies support the use of dexamethasone as a treatment in COVID-19 lower respiratory cases (180–182).

Remdesivir has received emergency use approval (EUA) by the Food and Drug Administration (FDA) on October 20, 2020. It is an antiviral treatment for adult patients and children from 12 years and above. Convalescent plasma was also approved as EUA by the FDA in August 2020. Bamlanivimab was approved for EUA by the FDA on November 9, 2020, and baricitinib was approved by the FDA as EUA on November 19, 2020 (183–185). Some researchers have suggested the use of renin-angiotensin system blockers in COVID-19 management. One hypothesis indicated that ACE2 receptor expression may increase with the use of ACE blockers, which may increase the severity of infections and manifestations. However, another study showed no difference in the outcome between the two groups of COVID-19 patients, wherein one group used an ACE blocker whereas the other one did not use an ACE blocker. The American Heart Association does not advise the initiation of ACE blockers and that its use should be stopped in COVID-19 patients (186, 187).

Chloroquine and hydroxychloroquine are no longer recommended in the management of COVID-19; the FDA revoked the EUA on June 15, 2020. Studies showed no significant effect between the two groups: one used chloroquine, while the other did not. Choloroquine is an antimalarial drug that can be used to control the inflammatory response in the body as SLE and rheumatoid arthritis; however, its use may induce a dangerous cardiac side effect (179, 188, 189).

Coronavirus disease-2019 patients may have coagulopathy that leads to microangiopathy, DVT, and PE. Antithrombotic therapy may be required in critically ill patients who are on mechanical ventilator support or they may require ICU admission to decrease mortality. Low-dose anticoagulants may prevent the deterioration of severe symptoms in hospitalized patients (190, 191).

Many COVID-19 patients developed new-onset diabetes mellitus as a result of pancreatic defects from COVID-19. Insulin should be used to treat diabetes in patients with COVID-19. Hospitalization is required for obese and chronic diabetes patients with COVID-19. The hypoglycaemic agent thiazolidinedione upregulates ACE2 receptors (33, 192, 193).

A British study showed a decrease in the mortality rate in patients who received low-dose dexamethasone 6 mg per day in comparison with other usual treatment patients. Corticosteroids may be recommended as a treatment for ARDS and septic shock. It is not useful in cardiogenic shock. Hydrocortisone 50 mg every 8 h per day can be useful in critically-ill COVID-19 patients (194–196).

Arrhythmia and cardiac manifestations in COVID-19 should be managed according to the guidelines of the European Resuscitation Council (ERC) and the American Heart Association (AHA) (67).

Other medications such as immunomodulators, including interleukin inhibitors and interferons, statins, and nitric oxide are still under study. Some natural supplements, such as zinc and vitamin D, are recommended (197–200).

Phosphodiesterase inhibitors, such as ibudilast, may be used as a macrophage migration inhibitor to manage the cytokine storm. It has been approved for use in Japan and South Korea for the treatment of bronchial asthma since 1989. In the USA, ibudilast has been approved by the FDA after trials (201).

Azithromycin and hydroxychloroquine are no longer recommended for use in the management of COVID-19. Studies showed no significant or clinical benefits in patients with hydroxychloroquine/azithromycin in comparison with other patients who did not use these drugs. Hydroxychloroquine/azithromycin may induce QT prolongation and cardiovascular mortality in COVID-19 patients (202, 203).

Empirical antibiotics are required for severe acute respiratory symptoms and septic symptoms (196).

## 4. Conclusion

COVID-19 is not only respiratory disease but also a multisystem disease, and respiratory symptoms may be present or absent in COVID-19 patients according to multiple factors such as viral overloading, genetic factors, immune reactions, cytokine storm, and comorbidities. The other frequent symptoms are gastrointestinal symptoms and fever. According to the severity of the case, the patient may also have renal, VTE, cardiac, or central nervous system manifestations. Skin and ocular manifestations have also been reported. In addition, peripheral neural disease was also reported in COVID-19 patients. The pathophysiology depends on ACE2 expression, cytokine storms, and side effects of the drugs used. The extra pulmonary manifestations of COVID-19 should not be neglected and should be fully considered for the early diagnosis and prevention of the spread of COVID-19.

## 5. Recommendations

We suggest changing the COVID-19 virus name from severe acute respiratory syndrome–Corona Virus two (SARS–CoV2) to severe respiratory and multisystem syndrome–Corona Virus Two (SRMS-CoV-2) or (SRAMS-CoV-2) because COVID-19 is not only respiratory disease but also a multisystem disease. We suggest that, in addition to the name change, extra pulmonary manifestations should not be ignored by healthcare providers and should be considered during the diagnosis and management of COVID 19.

## Author Contributions

This article was completed in partial requirement for the MSc in Acute Medicine from the University of South Wales, UK. IE was the student and data collector. KN was a supervisor and tutor. Both authors contributed to the article and approved the submitted version.

## Conflict of Interest

The authors declare that the research was conducted in the absence of any commercial or financial relationships that could be construed as a potential conflict of interest.

## Publisher's Note

All claims expressed in this article are solely those of the authors and do not necessarily represent those of their affiliated organizations, or those of the publisher, the editors and the reviewers. Any product that may be evaluated in this article, or claim that may be made by its manufacturer, is not guaranteed or endorsed by the publisher.
